# Uremia Impacts VE-Cadherin and ZO-1 Expression in Human Endothelial Cell-to-Cell Junctions

**DOI:** 10.3390/toxins10100404

**Published:** 2018-10-07

**Authors:** Rayana A. P. Maciel, Regiane S. Cunha, Valentina Busato, Célia R. C. Franco, Paulo C. Gregório, Carla J. R. Dolenga, Lia S. Nakao, Ziad A. Massy, Agnès Boullier, Roberto Pecoits-Filho, Andréa E. M. Stinghen

**Affiliations:** 1 Experimental Nephrology Laboratory, Basic Pathology Department, Universidade Federal do Paraná, Curitiba 80050-540, Brazil; rayanaariane@gmail.com (R.A.P.M.); regidacunha@gmail.com (R.S.C.); valentinabusatoo@gmail.com (V.B.); paulocezargregorio@gmail.com (P.C.G.); 2 Cell Biology Department, Universidade Federal do Paraná, Curitiba 80050-540, Brazil; crcfranc@ufpr.br; 3 Basic Pathology Department, Universidade Federal do Paraná, Curitiba 80050-540, Brazil; carla.dolenga@ufpr.br (C.J.R.D.); lia.nakao@ufpr.br (L.S.N.); 4 Division of Nephrology, Ambroise Paré University Hospital, APHP, Boulogne-Billancourt, 92100 Paris, France and Inserm U1018, Team 5, CESP, UVSQ, Paris-Saclay University, 94800 Villejuif, France; ziad.massy@aphp.fr; 5 Universitè de Picardie Jules Verne, MP3CV and CHU d’Amiens, 80025 Amiens, France; agnes.boullier@u-picardie.fr; 6 Pontifícia Universidade Católica do Paraná, School of Medicine, Curitiba 80215-901, Brazil; r.pecoits@pucpr.br

**Keywords:** chronic kidney disease, uremic toxins, intercellular junctions

## Abstract

Endothelial dysfunction in uremia can result in cell-to-cell junction loss and increased permeability, contributing to cardiovascular diseases (CVD) development. This study evaluated the impact of the uremic milieu on endothelial morphology and cell junction’s proteins. We evaluated (i) serum levels of inflammatory biomarkers in a cohort of chronic kidney disease (CKD) patients and the expression of VE-cadherin and Zonula Occludens-1 (ZO-1) junction proteins on endothelial cells (ECs) of arteries removed from CKD patients during renal transplant; (ii) ECs morphology in vitro under different uremic conditions, and (iii) the impact of uremic toxins p-cresyl sulfate (PCS), indoxyl sulfate (IS), and inorganic phosphate (Pi) as well as of total uremic serum on VE-cadherin and ZO-1 gene and protein expression in cultured ECs. We found that the uremic arteries had lost their intact and continuous endothelial morphology, with a reduction in VE-cadherin and ZO-1 expression. In cultured ECs, both VE-cadherin and ZO-1 protein expression decreased, mainly after exposure to Pi and uremic serum groups. VE-cadherin mRNA expression was reduced while ZO-1 was increased after exposure to PCS, IS, Pi, and uremic serum. Our findings show that uremia alters cell-to-cell junctions leading to an increased endothelial damage. This gives a new perspective regarding the pathophysiological role of uremia in intercellular junctions and opens new avenues to improve cardiovascular outcomes in CKD patients.

## 1. Introduction

The constant endothelium aggression resulting from the interaction between uremic toxins and endothelial cells (ECs), with cellular phenotype alteration, may result in elevated plasma levels of vascular inflammatory biomarkers, such as IL-8 and MCP-1 (CCL2) cytokines and adhesion molecules VCAM-1 and ICAM-1 [1]. Serum levels of these biomarkers increase during advanced chronic kidney disease (CKD) stages, suggesting a link between vascular activation, inflammation and uremic toxicity [2,3].

Uremic toxins, such as p-cresyl sulfate (PCS), indoxyl sulfate (IS) and inorganic phosphate (Pi), are frequently related to endothelium dysfunction found in CKD patients. Consequently, the uremic toxins can induce active free radicals [4], endothelial microparticle production [5], and cytoskeletal remodeling, while resulting in increased permeability, interfering with the intercellular junctions [6,7].

Endothelial cell junctions such as tight junctions (TJs), adherent junctions (AJs), and gap junctions (GJs) control important functions in cell homeostasis [8]. In uremia, these cellular junctions may be impaired, which can favor endothelial injury and progressive cardiovascular disease [9,10]. It was demonstrated that VE-cadherin, an AJ, is necessary for endothelial integrity in quiescent vessels and the correct organization of new vessels [11]. Some accessory molecules are important in regulating VE-cadherin in the cytoplasmic domain, like p120-catenin (p120), β-catenin and vinculin [12,13,14]. Studies involving Zonula Occludens-1 (ZO-1), a TJ protein, have shown that in ZO-1 deficient cells, there is a delay in the recruitment of other TJ proteins, indicating that ZO-1 plays an important role in the regulation of other TJs [15]. ZO-1 is also a central regulator of VE-cadherin-dependent endothelial junctions that organize the cytoskeleton, tune cell-to-cell tension, migration, angiogenesis, and barrier formation [16].

The present study aims at investigating systematically the effect of uremia on the expression of VE-cadherin and ZO-1 in endothelial cell junctions, to better understand the pathophysiological role of uremia in disruption of intercellular junctions and cardiovascular impact, thus, allowing us to gain new perspectives, leading to the discovery of novel therapeutic strategies, that might help in preventing the development of cardiovascular complications in CKD patients.

## 2. Results

### 2.1. Clinical and Laboratory Characteristics of the Study Population

Clinical and laboratory characteristics of the study population are shown in Table 1 and Table 2.

The median eGFR (CKD-EPI) was 44.47 mL/min (range 7.20–157.30 mL/min). Patients who were taking antihypertensives (84%), anticoagulants (44%), diuretics (55%), antidiabetics (40%), statins (49%), and phosphate binders (6%).

### 2.2. Clinical and Biochemical Characteristics of Each Uremic Pool

For in vitro experiments, we prepared three pools of uremic serum (GI, GII, and GIII) according to their uremia levels. Patients were distributed according to CKD stages 1 to 5 of Kidney Disease: Improving Global Outcomes (KDIGO). Among the 80 patients, 8.75% (*n* = 7) were classified as mild CKD (GI-stage 1), 52.50% (*n* = 42) in moderate CKD (GII-stages 2 and 3), and 38.75% (*n* = 31) in severe CKD (GIII-stages 4 and 5) (Table 3).

### 2.3. Concentration of Systemic and Vascular Inflammatory Biomarkers

The median values of MCP-1, IL-8, sVCAM-1, and sICAM-1 in CKD patients serum were 210.9 pg/mL, 37.0 pg/mL, 484.1 ng/mL and 532.3 ng/mL, respectively. There was a significant correlation between MCP-1 vs. sVCAM-1 (*P* < 0.01; ρ = 0.28), MCP-1 vs. eGFR (*P* < 0.001; ρ = −0.29), sICAM-1 vs. sVCAM-1 (*P* < 0.01; ρ = 0.54), sVCAM-1 vs. eGFR; *P* < 0.0001; ρ = −0.41) and hs-CRP vs. eGFR (*P* < 0.01; ρ = −0.29).

### 2.4. Multivariate Analysis of Independent Determinants of Chemokines, Adhesion Molecules

In this multivariate analysis model, eGFR is independently associated with circulating levels of MCP-1 (*P* < 0.001), sVCAM-1 (*P* < 0.0001), hs-CRP (*P* < 0.01), and hypertension (*P* < 0.01). sVCAM-1 levels were associated with Diabetes mellitus (*P* < 0.05) and hs-CRP levels were associated with Diabetes mellitus and hypertension (*P* < 0.05).

### 2.5. Correlations between Uremic Toxins Serum Concentration and eGFR

**Figure 1:**
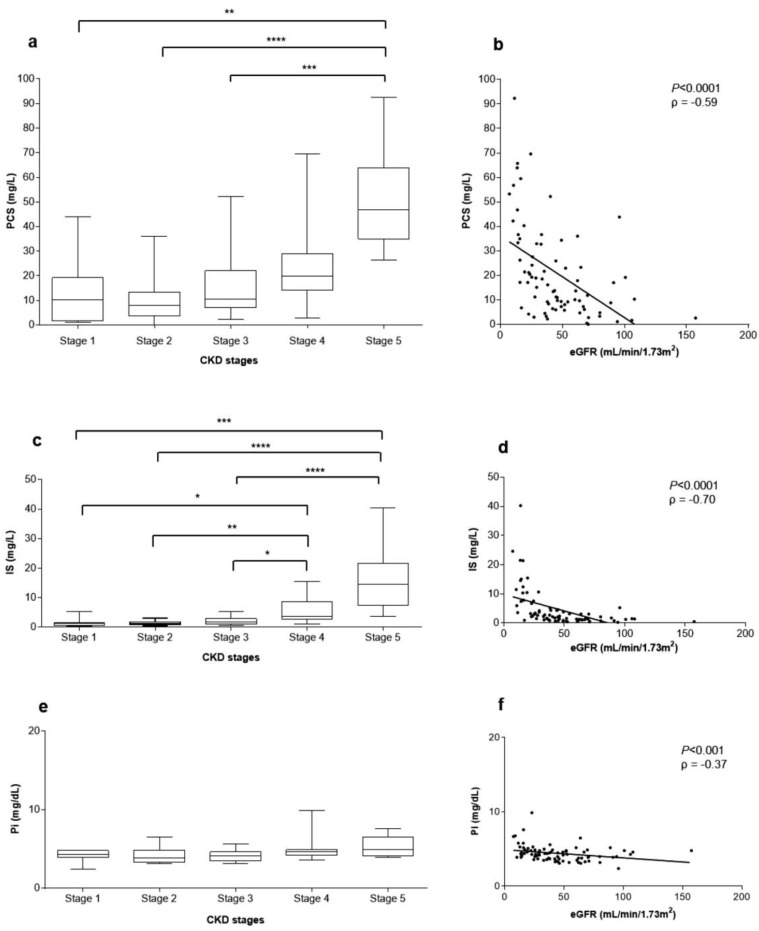
Uremic toxins’ serum concentrations and correlation with eGFR. Right panel. Box plots of the p-cresyl sulfate (PCS) (**a**), indoxyl sulfate (IS) (**c**) and inorganic phosphate (Pi) (**e**) serum concentrations in patients’ according to their chronic kidney disease (CKD) stages. Left panel. Correlation between PCS (**b**), IS (**d**), Pi (**f**) serum concentrations and eGFR in CKD patients (**** *P* < 0.0001, ρ = −0.59; *** *P* < 0.0001, ρ = −0.70; ** *P* < 0.001, ρ = −0.37, respectively).

The median concentration of uremic toxins PCS, IS, and Pi in patients serum were 39.79 mg/L, 4.59 mg/L, and 4.4 mg/dL, respectively. There was a significant correlation between serum concentrations of PCS vs. IS (*P* < 0.0001) and PCS vs. Pi (*P* < 0.0001). Contrarily, there was no significant correlation between IS vs. Pi. PCS, IS, and Pi serum concentration distributions according to CKD stages in pre-dialysis patients are shown in Figure 1a,c,e. Figure 1b,d,f show that PCS, IS, and Pi serum concentrations were significantly and inversely correlated with eGFR (*P* < 0.0001, ρ = −0.59; *P* < 0.0001, ρ = −0.70, and *P* < 0.001, ρ = −0.37, respectively) (Figure 1).

### 2.6. VE-Cadherin and ZO-1 Expression Increased in CKD Iliac and Renal Arteries

**Figure 2:**
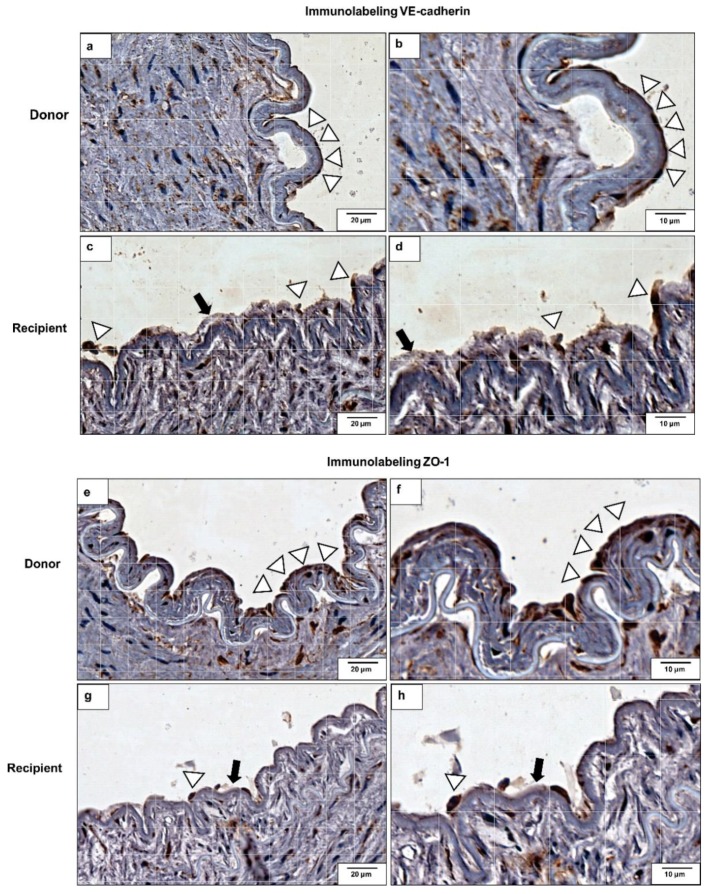
VE-cadherin and Zonula Occludens-1 (ZO-1) protein expressions in renal arteries. VE-cadherin immunolabeling in renal artery of (**a**,**b**) donor (control) and (**c**,**d**) recipient (CKD patient). ZO-1 immunolabeling in renal artery of (**e**,**f**) donor (control) and (**g**,**h**) recipient (CKD patient). Magnifications: 100× (**a**,**c**,**e**,**g**) and 400× (**b**,**d**,**f**,**h**). Arrowheads indicate intact endothelial cell monolayer. Arrows indicate loss of endothelial monolayer’s integrity. Positive immunoreaction was observed as a brown precipitate. Photos shown are representative of all the analyses.

For investigating in vivo VE-cadherin and ZO-1 protein expression, we performed an immunolabeling on iliac and renal arteries from donors (controls) and from CKD recipients (Figure 2). In the donors’ arteries sections, an intact and continuous endothelium is observed with strong VE-cadherin (Figure 2a,b) and ZO-1 (Figure 2e,f) labeling. On the other hand, endothelial cell monolayer breakdowns, characteristic of endothelial injury and structural damage, were observed in the recipients’ arteries sections as shown by a decrease in VE-cadherin (Figure 2c,d) and ZO-1 (Figure 2g,h) immunolabeling.

### 2.7. Cell Viability

**Figure 3:**
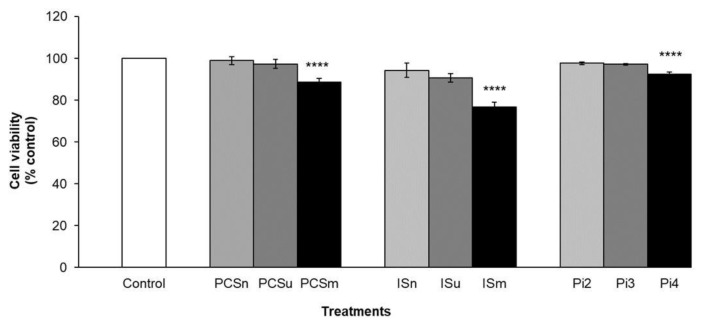
Effect of PCS, IS and Pi on cell viability. Cells were incubated with the various concentrations of uremic toxins or serum for 24 h. Cell viability was assessed using the MTT (3-(4,5-Dimethylthiazol-2-yl)-2,5-diphenyltetrazolium bromide) method. Control (non-treated cells); PCS normal (PCSn) and IS normal (ISn) at normal concentrations; PCS uremic (PCSu) and IS uremic (ISu) at minimum uremic concentrations; PCS maximum uremic (PCSm) and IS Maximum uremic (Ism) at maximal uremic concentrations; inorganic phosphate at 2 mM (Pi2), 3 mM (Pi3), and 4 mM (Pi4). Results are expressed as % of control (non-treated cells). **** *P* < 0.0001 for PCSm, Ism, and Pi4 vs. control.

Cell viability significantly decreased in cells treated with PCS maximum uremic (PCSm), Is maximum uremic (Ism), and Pi4 (*P* < 0.0001) when compared to control (non-treated-cells) (Figure 3). However, cell treatment with the uremic pools (GI, II, and III) induced no change in cell viability when compared to control (non-treated cells).

### 2.8. Uremic Milieu Increases Endothelial Cell Permeability

**Figure 4:**
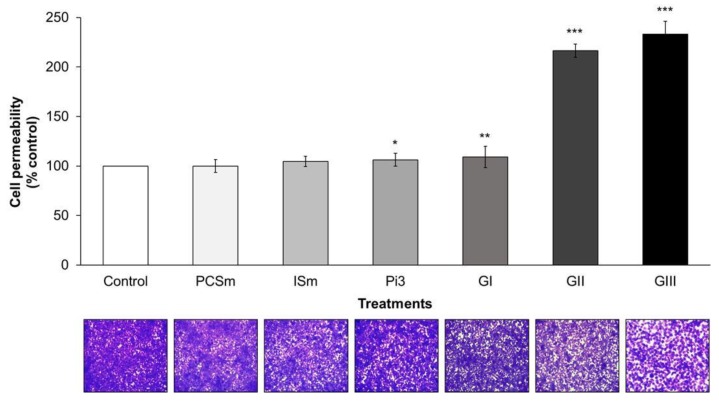
Effect of uremic toxins and uremic pools on cell permeability. Endothelial cells were seeded at high density and cultured until confluency. Cells were then exposed to the various concentrations of uremic toxins or 10% of uremic pools. Fluorescein isothiocyanate (FITC)-conjugated dextran (4 kDa, final concentration 2.5 mg/mL) was added to the upper compartment of the inserts for 20 min. Aliquots were then taken from the lower compartment and fluorescence read. Permeability is expressed as % of control (non-treated cells) and represents mean ± SEM (standard mean error) of 12 determinations. * *P* < 0.05 Pi3 vs. control; ** *P* < 0.01 GI vs. control; *** *P* < 0.001 GII and GIII vs. control. Below are shown the crystal violet staining of the cells in the corresponding inserts.

Cell permeability was evaluated after cell treatment with uremic toxins at their maximum uremic concentrations (PCSm, ISm), with Pi3 and with 10% uremic pools (GI, II, and III). Paracellular permeability was assessed by measuring the passage of Fluorescein isothiocyanate (FITC)-dextran through the endothelial monolayer. There was a significant increase in the cellular permeability of cells treated with Pi3 (*P* < 0.05), GI (*P* < 0.01), GII, and III (*P* < 0.001), compared to control (non-treated cells) (Figure 4). These results were confirmed by the visualization of the endothelial monolayer on the transwell inserts stained with crystal violet (Figure 4, lower panel).

### 2.9. Uremia Impacts the Intercellular Adhesion and the Endothelial Cell Phenotype

**Figure 5:**
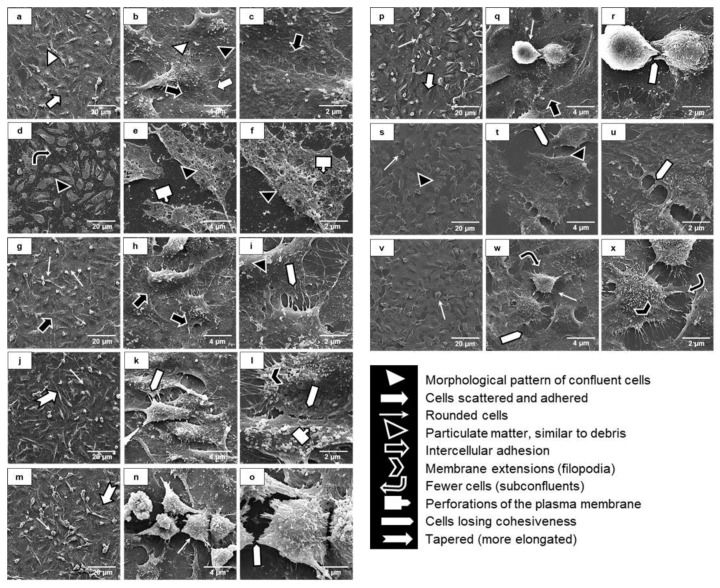
Morphological and ultrastructural analyses of endothelial intercellular junctions by scanning electronic microscopy (SEM) Non-treated cells were used as negative controls (**a**–**c**). 4% dimethyl sulfoxide (DMSO)-treated cells were used as positive controls (**d**–**f**). Cells were treated with PCSm (**g**–**i**), ISm (**j**–**l**), Pi3 (**m**–**o**), GI (**p**–**r**), GII (**s**–**u**), and GIII (**v**–**x**) for 24 h. Magnifications of the images: **a**,**d**,**g**,**j**,**m**,**p**,**s** (1000×), **b**,**e**,**h**,**k**,**n**,**q**,**t**,**w** (6000×), **c**,**f**,**i**,**l**,**o**,**r**,**u**,**x** (15,000×).

The morphological and ultrastructural patterns of an intact endothelial monolayer are shown in Figure 5a–c with non-treated human endothelial cells (negative control). We used 4% dimethyl sulfoxide (DMSO)-treatment, known to induce endothelial monolayer breakdowns, as the positive control (Figure 5d–f), which show several morphological and ultra-structural changes. Majority of cells exposed to PCSm (Figure 5g–i), were rounded, with a shrunken cell body. The cells exposed to ISm (Figure 5j–l) presented an elongated morphology with long tapered expanses of the cell body. Treatment with Pi3 (Figure 5m–o) induced a loss of the cell’s capacity to adhere and spread. Altogether, these results show that among the three toxins tested, Pi was the one that induced the greatest loss of intercellular adhesion and altered their morphological and ultrastructural pattern. Cells were also exposed to different uremic serum pools. The morphological and ultrastructural pattern remained unchanged when the cells were incubated with GI (Figure 5p–r). However, cells treated with either GII (Figure 5s–u) or GIII (Figure 5v–x) demonstrated intercellular loss of cohesiveness and a smaller number of cells, mostly rounded and less spread onto the substrate (Figure 5).

### 2.10. Uremic Environment Modifies the Endothelial Cell Cytoskeleton

**Figure 6:**
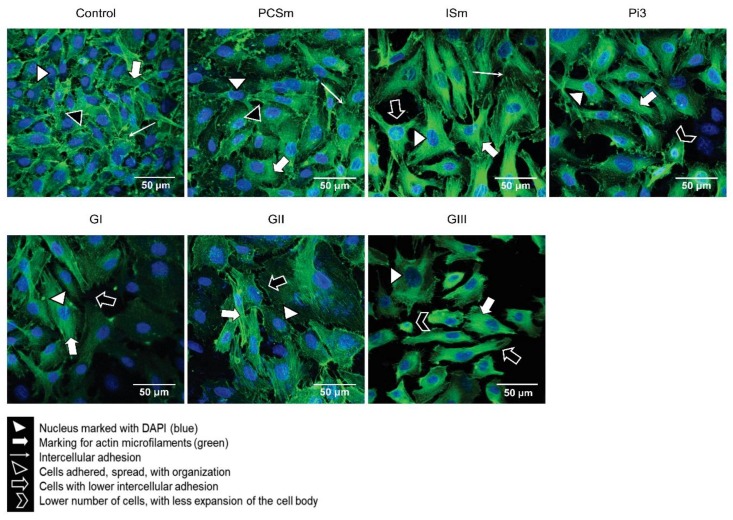
Effect of a uremic environment on F-actin protein expression. After treatment with either uremic toxins or uremic pools, F-actin protein expression was visualized using ActinGreen™ 488 ReadyProbes^®^ (green). DAPI (4′,6-diamidino-2-phenylindole) was used to label the nuclei (blue). Control (non-treated cells), Magnification 600×.

Direct association of the actin cytoskeleton with cell adhesion proteins is essential in the organization of adherens junctions and, therefore, barrier function. The cells treated with PCSm showed the same pattern as the control cells, with cells adhered and scattered, with a central nucleus and an organized actin cytoskeleton, maintaining intercellular adhesion. The cells treated with ISm, were less juxtaposed and more elongated, with a more expanded length of the cell body. For cells treated with Pi3, a smaller number of cells, with a lower cell body expansion and characteristic of suffering cells, less juxtaposition and distinct morphologies, were observed. Cells treated with GI and GII were partially confluent and had distinct morphologies. The cells treated with Pi3 and GIII showed greater impairment in the degree of adhesion, spreading and juxtaposition (Figure 6).

### 2.11. Uremic Environment Impacts Endothelial Cell Adherent Junction and VE-Cadherin Expression

**Figure 7:**
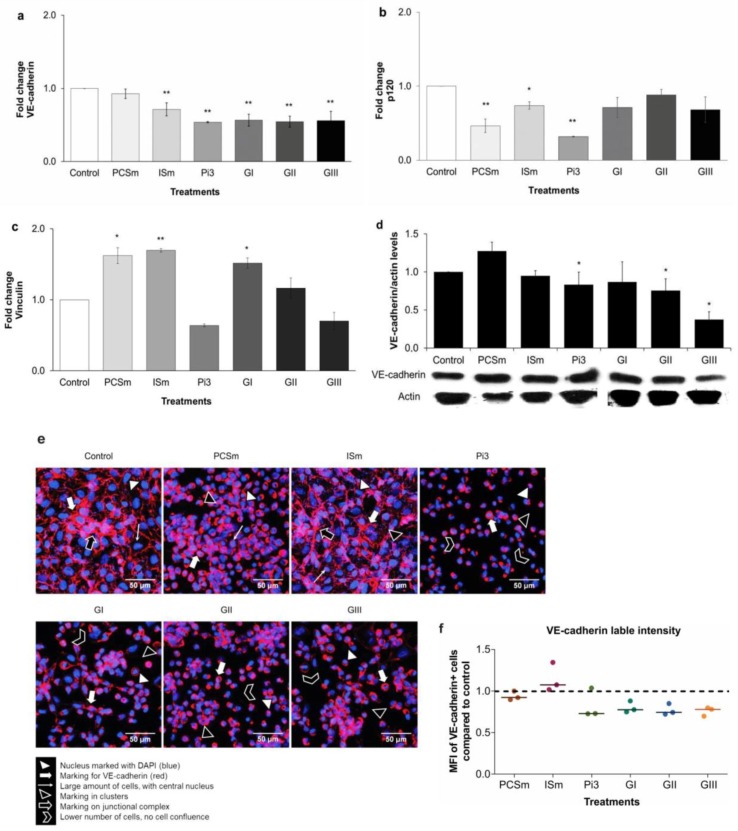
Uremic environment impacts endothelial cell adherent junctions and VE-cadherin expression. Control (non-treated cells). Effect of uremic toxins and uremic pools on VE-cadherin, p120 and vinculin gene expression: VE-cadherin-** *P* < 0.01 for ISm, Pi3, GI, GII, and GIII vs. Control (**a**); p120-** *P* < 0.01 for PCSm and Pi3 vs. Control (**b**); vinculin-* *P* < 0.05 for PCSm and GI vs. Control and ** *P* < 0.01 for ISm vs. Control (**c**). Effect of uremic toxins and uremic pools on VE-cadherin protein expression: by immunoblotting: Actin was used as protein loading control. Lower panel: A representative immunoblot of 8 experiments. Upper panel: Quantification of the bands (**d**), by immunofluorescence staining: Magnification 600× (**e**) and by flow cytometry: Fluorescence intensity was evaluated by flow cytometry and quantified using Flowing 2 software. Dots represent median fluorescence intensity (MFI) compared to control (non-treated cells; dashed lines) in each experiments (*n* = 6). Bars represent median of all values for each group (**f**).

We investigated gene expression of VE-cadherin, p120, vinculin, and β-catenin (Figure 7). For VE-cadherin, a significant decrease in gene expression in cells treated with ISm and Pi3 was observed (*P* < 0.01) (Figure 7a). In addition, there was a significant decrease in p120 gene expression in cells treated with PCSm (*P* < 0.01), ISm (*P* < 0.05 and Pi3 (*P* < 0.01) (Figure 7b). PCSm and GI increased vinculin gene expression (*P* < 0.05), as well as ISm (*P* < 0.01) (Figure 7c). Conversely, β-catenin gene expression remained unchanged irrespective of the treatment. The decrease in the VE-cadherin gene expression at the protein level was confirmed by Western blot analysis. Our results showed a decrease in the VE-cadherin protein expression only in cells treated with Pi (*P* < 0.05) (Figure 7d). Cells were also treated with the different uremic sera (GI, GII, GIII). As shown in Figure 7a, only VE-cadherin gene expression significantly decreased with the three pools (*P* < 0.01), while the p120 and vinculin gene expression remained unaffected. Treatment with GII or GIII was also able to decrease VE-cadherin protein expression (*P* < 0.05). To quantify and demonstrate the localization of VE-cadherin, we performed immunofluorescence staining (Figure 7e) and flow cytometry (FACS) (Figure 7f). In cells treated with PCSm and Ism, a large numbers of confluent cells similar to control, were observed. Contrarily, cells treated with Pi, GI, GII, and GIII, demonstrated similar morphological patterns, and were observed as clusters (Figure 7e). FACS showed approximately 30% decrease in the labeling intensity of Pi3-treated cells, with all uremic pools (Figure 7f). Altogether, these data demonstrate that uremia has a significant impact on the VE-cadherin gene and protein expression, while also affecting the associated adherent protein junctions.

### 2.12. Uremic Environment Differently Modulates ZO-1 Gene and Protein Expression

**Figure 8:**
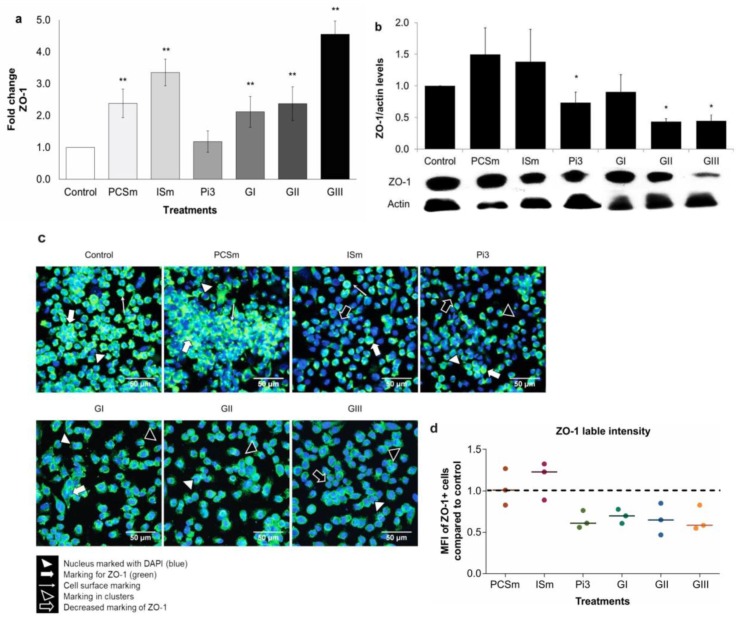
Uremic environment differently modulates ZO-1 gene and protein expression. Control (non-treated cells). Effect of uremic toxins and uremic pools on ZO-1 gene expression. ** *P* < 0.01 for PCSm, ISm, GI, GII, and GIII vs. Control (**a**); Effect of uremic toxins and uremic pools on ZO-1 protein expression by Western blotting: Actin was used as a loading control. Lower panel: A representative immunoblot of 8 experiments. Upper panel: Quantification of the bands. * *P* < 0.05 for Pi3, GII and GIII vs. Control; (**b**), by immunofluorescence: After treatment, cells were labeled for ZO-1 (green) and nuclei (blue) (magnification 600×) (**c**) and by flow cytometry: Fluorescence intensity was evaluated by flow cytometry and quantified using Flowing 2 software. Dots represent median fluorescence intensity (MFI) as compared to Control (dashed lines) in each experiment (*n* = 6). Bars represent median of all values for each group (**d**).

To study the role of uremic environment on tight junction disruption and ZO-1 gene and protein expression, we treated ECs with toxins at their maximum uremic concentrations (PCSm, ISm), Pi at 3 mM and 10% of uremic serum (GI, GII and GIII) (Figure 8). There was a significant increase in ZO-1 gene expression, (*P* < 0.01) in cells treated with PCSm, ISm, GI, GII, and GIII (Figure 8a). No significant change was observed in presence of Pi3. Remarkably, at the protein level, ZO-1 expression significantly decreased in cells treated with Pi3, GII, and GIII (*P* < 0.05) (Figure 8b), possibly indicating a post-transcriptional regulation of this protein. The immunofluorescence staining showed that the cells treated with PCSm and ISm had a more intense labeling pattern, on the surface, mainly in aggregated areas (clusters), which was not found in control cells (non-treated cells). Flow cytometry analysis showed up to 35% decrease in ZO-1 labeling, in the presence of Pi3, GI, GII, and GIII (Figure 8c).

## 3. Discussion

The main findings of the present study are: (I) Uremia impacts the cellular adhesion and disturbs the homeostasis of endothelial cell phenotype; (II) Uremic environment modifies the endothelial cell cytoskeleton and F-actin protein expression; (III) Uremic environment impacts endothelial cell adherent junction and VE-cadherin expression, and (IV) Uremic environment compromises tight junction and differently modulates ZO-1 gene and protein expression. To our knowledge, our study is the first to evaluate in vitro the effect of uremic toxins, especially uremic serum on the cell junctions in human endothelial cells.

This study comprised 80 patients in various stages of CKD, predominantly elderly, male, and Caucasian, with typical CKD clinical characteristics. The serum levels of systemic and vascular inflammatory biomarkers found, such as hs-CRP, MCP-1, IL-8, sVCAM-1, and sICAM-1, are also in accordance with similar studies, which showed that inflammation is strongly correlated with CKD [3,17,18]. We observed a significant correlation between MCP-1, sVCAM-1, and hs-CRP with eGFR and hypertension, corroborating previous studies [19]. Indeed, these vascular and inflammatory biomarkers are highly expressed in CKD patients, being intimately associated with endothelium dysfunction and cardiovascular diseases (CVD) [3,20] as well intima/media compromising, as recently demonstrated by our group [2,3,21].

Further corroborating previous studies, our data demonstrated higher serum levels of PCS, IS, and Pi in patients with severe CKD compared to the initial and moderate stages of CKD [22]. It was demonstrated that high PCS serum levels induce the release of endothelial cell microparticles [23], reactive oxygen species (ROS) activation by leukocytes [4], and vascular remodeling [24]. Clinical studies associated high PCS serum levels with diabetic nephropathy and CVD [25], besides PCS, has been considered a predictor of mortality in patients at different stages of CKD [26,27]. Similarly, high IS serum concentrations lead to endothelial injury and triggers the production of pro-inflammatory molecules, which in turns inhibit endothelial regeneration and repair [28]. Studies demonstrated that IS may contribute to cerebral endothelium dysfunction in CKD patients due to increased oxidative stress [29]. In vivo studies have also shown that IS is correlated with vascular dysfunction associated with CKD [30] and promotes aortic calcification [31]. Conversely, although we found high Pi serum levels in more severe CKD, there was no significant difference between the uremic groups. One hypothetic explanation is that hyperphosphatemia is prevented until the later stages of CKD by parathyroid hormone (PTH) [32] and fibroblast growth factor-23 (FGF-23) [33], which in turn suppress renal phosphate reabsorption and augment renal phosphate excretion in these patients [34].

First, we demonstrated that there was a significant increase in cell permeability, suggesting junction disruption mainly in cells treated with Pi3 and uremic pools at different uremia levels. Uremic serum can extensively disturb cell-to-cell junctions, which can be contributed to the fact that uremic serum is composed of more than 150 toxins (EUTox, 2018), acting together, often synergistically, which damage the systemic endothelium and consequently increase the intensity of the inflammatory process [29]. In fact, Peng et al. (2012) showed that after IS treatment, the permeability of pulmonary endothelial cells increased, through cytoskeletal remodeling and cell contraction, which resulted in disruption of the intercellular junctions [6]. Validating our findings through scanning electronic microscopy (SEM) and F-actin immunostaining, it was observed that uremic serum and high Pi concentrations severely altered endothelial cell morphology, compromising the cytoskeleton. It was demonstrated that uremia disorganizes the cytoskeleton of platelets [35] and modulates the phenotype of smooth muscle cells [36,37]. An intact and continuous endothelium with well-labeled VE-cadherin and ZO-1 was observed in the donors’ arteries. In contrast, recipients’ arteries showed endothelium monolayer failures, characteristic of injury and structural damage. This loss of endothelial barrier integrity occurs due to the disruption of intercellular junctions [9,38].

The expression of endothelial cell proteins, from the adherent junctions (AJ), i.e., VE-cadherin, p120, β-catenin, and vinculin was investigated. Our data demonstrated a significant decrease in VE-cadherin gene and protein expressions in cells treated with IS, Pi, and uremic pools. Similarly, previous studies have demonstrated that VE-cadherin silencing leads to loss of vascular integrity [39]. Indeed, it was demonstrated that VE-cadherin gene knockout was lethal in mouse embryos due to angiogenic defects related to endothelial apoptosis [40]. VE-cadherin concentration can also be used as a biomarker of endothelial dysfunction associated with coronary artery disease, confirming the involvement of this protein in the development of CVD [41]. Studies have shown that Vascular endothelial growth factor (VEGF), a protein associated with inflammatory biomarkers in CKD patients [42], is capable of inducing VE-cadherin phosphorylation, which leads to its subsequent endocytosis and degradation in angiogenesis [43]. Furthermore, we evaluated the expression of other proteins involved in adherent junction, such as p120, β-catenin, and vinculin. However, our results demonstrated that the mRNA of these proteins is only affected by toxins treatment and not by the presence of uremic sera.

Treatment of ECs with PCS, IS, and uremic serum significantly increased ZO-1 gene expression, whereas that with Pi decreased. On the other hand, ZO-1 protein expression was decreased in cells treated with Pi and uremic serum (moderate and severe CKD). These results are in accordance with previous studies that evaluated the damage to the gut barrier integrity in a CKD model [44,45,46,47]. In fact, these authors analyzed ZO-1 expression in the colonic tissue of nephrectomized rats and observed an increased gene expression and a decrease in protein levels [44,45]. This difference between ZO-1 gene and protein expression may be due to post-transcriptional or post-translational regulatory mechanisms. Similarly, other studies [46,47] demonstrated that urea and uremic serum of CKD patients significantly reduced the protein expression of ZO-1 and others tight junction’s proteins in colonocytes. According to literature, the family ZO proteins carry domains required for structural organization of intercellular junctions and additional domains capable of functioning in signal transduction pathways [48]. Therefore, the uremic environment could, hypothetically, affect, at least in part, the regulatory functional capacity of ZO-1 [49].

In summary, the mechanisms by which uremia alters VE-cadherin and ZO-1 expression, according to the literature, could be several including (I) VE-cadherin is required for endothelial integrity in quiescent vessels and for correct organization of new vessels [1], and the rapid transient phosphorylation of VE-cadherin in endothelial cells can be induced by vascular endothelial growth factor (VEGF). (II) Some accessory molecules are important in regulating VE-cadherin in the cytoplasmic domain, such as β-catenin, p120-catenin (p120), and vinculin. These interactions regulate the association of cadherins with the actin cytoskeleton and are important for strong cell–cell adhesion. In addition P120 suppresses cadherin endocytosis and regulates the cytoskeleton [2,3]. Dephosphorylation of β-catenin together with VE-cadherin contributes to the stabilization of the endothelial cell–cell junction. However, β-catenin may exert broader effects on gene expression and also bind to cadherins in cell-to-cell AJs and stabilize their interaction with the cytoskeleton [4]. The vinculin protects the VE-cadherin junctions from the aperture during force-dependent remodeling [5] and is an important regulator of cell–cell junctions [6,7]. (III) Studies involving Zonula Occludens-1 (ZO-1) demonstrate that ZO-1 plays an important role in the regulation of other TJs [8]. ZO-1 is also an important central regulator of VE-cadherin-dependent endothelial junctions, which organizes the cytoskeleton, adjusting cell–cell tension, migration, angiogenesis, and barrier formation [9].

We recognize that our study has some limitations, and further work will be required. First, there are multiple external variables comparisons, due to patients’ clinical and biochemical parameters, that generates a panorama difficult to control, which could influence the results. Second, the evaluation of the effect of uremia on VE-cadherin phosphorylation and its internalization, as well the extensive involvement of p120, β-catenin, vinculin proteins, besides elucidating the ZO-1 protein regulation mechanisms, should be deeply investigated. Our study opens perspectives to evaluate the involvement of innumerable intercellular junction proteins in order to better elucidate the relationship of uremia with changes in endothelial injury and vascular permeability.

## 4. Conclusions

In conclusion, this study was the first to demonstrate at different levels of response, namely cell, tissue and circulation that uremia impacts the endothelial cell-to-cell junctions, and the more advanced the CKD, the greater is the endothelial damage. The impact on VE-cadherin and ZO-1 is more significant in moderate and severe stage CKD and is distinct when analyzing the uremic toxins alone or the uremic sera, which suggests different mechanisms of each toxin, and/or a synergistic mechanism. In vitro, we demonstrated that VE-cadherin gene and protein expression are decreased in more advanced CKD. Despite of an increase in ZO-1 gene expression, the ZO-1 protein levels decreased, suggesting a possible role of ZO-1, in the regulation of other cell junction proteins.

## 5. Materials and Methods

To better understand the methodology design, please verify the flowcharts on supplementary materials available (Figures S1–S3).

### 5.1. Patients

Experiments were conducted as per the Ethical Committee of Federal University of Paraná (Curitiba, Brazil) and Ethical Committee of Pontifical Catholic University of Paraná (Curitiba, Brazil), under the numbers CEP/SD-PB nº 980959 (11/03/2015) and CEP/PUC-PR nº 557 (26/06/2005), respectively. Informed consent was obtained from the patients. For this study, peripheral blood samples were collected from 80 patients from a CKD clinic at Pontifícia Universidade Católica do Paraná (Curitiba, Brazil) (March-August, 2015). Inclusion criteria were: age between 18–80 years, presence of CKD, and willingness to participate in the study. Exclusion criteria were signs of active inflammatory or infectious diseases, malignancy, previous or current renal function therapy, autoimmune diseases, neoplasms, hepatic dysfunction, and use of immunosuppressive drugs in the last three months before inclusion in the study. The patients’ glomerular filtration rates (eGFR) were estimated by the CKD-EPI (Chronic Kidney Disease Epidemiology Collaboration) [50].

#### 5.1.1. Patients’ Samples Collection and Processing for In Vivo and In Vitro Assays

Serum was collected, divided into aliquots, and stored at −80 °C. Patients were classified into stages 1–5 according to the Kidney Disease: Improving Global Outcomes (KDIGO) recommendations [51]. For in vitro experiments, three pools of serum from CKD patients were prepared as follows: Group I: Patients with latent CKD (GI, stage 1; *n* = 7), Group II: Patients with mild CKD (GII, stages 2 and 3; *n* = 42) and Group III: Patients with severe CKD (GIII, stages 4 and 5; *n* = 31).

#### 5.1.2. Clinical and Biochemical Characteristics of the Patients

During inclusion, the patients were characterized according to their age, gender, race, smoking, presence of Diabetes mellitus and/or hypertension. High sensitivity C-reactive protein (hs-CRP) was assessed by an immuno-turbidimetric assay (AU680, Beckman Counter, CA, USA) and others biochemical parameters were measured by colorimetric methods (Vitros 5,1 Fusion, Ortho Clinical Diagnostics, NJ, USA).

### 5.2. Materials

Dulbecco’s Modified Eagle Medium (DMEM), Fetal Bovine Serum (FBS), penicillin/streptomycin were purchased from Gibco (Grand Island, NY, USA). Anti-human MCP-1, IL-8, sVCAM-1, and sICAM-1 antibodies, mouse anti-human VE-cadherin monoclonal antibody, and NorthernLights™ 557 conjugated anti-mouse IgG secondary antibody were obtained from R&D Systems (Minnesota, MN, USA). The Transwell^®^ inserts were from Corning (New York, NY, USA). ActinGreen™ 488 ReadyProbes^®^, Trizol, primers, PVDF membranes, and rabbit anti-human ZO-1 polyclonal antibody were purchased from Invitrogen (Carlsbad, CA, USA). Fluoromount G and DAPI were obtained from Life Technologies (Carlsbad, CA, USA). Horseradish peroxidase-conjugated goat anti-mouse IgG, goat anti-rabbit IgG antibodies, and Alexa Fluor 488 secondary antibody were purchased from Thermo Fisher (Massachusetts, MA, USA). All other reagents were obtained from Sigma-Aldrich (St. Louis, MO, USA) if not otherwise specified.

### 5.3. Uremic Toxins’ Preparation

PCS was synthetized by p-cresol (PC) sulfation and characterized as previously described by Feigenbaum and Neuberger (1941), with slight modifications [2,52,53]. For the concentrations used in our experiments, we to referred the list of uremic toxins provided by the European Uremic Toxin Work Group (EUTox, http://eutoxdb.odeesoft.com/index.php). PCS and IS were used at normal (n) (0.08 and 0.6 mg/L), uremic (u) (1.75 and 53 mg/L) and maximum uremic (m) (2.6 and 236 mg/L) [22,54] concentrations, respectively. A solution of Na2HPO4.7H2O, used as a source of Pi, was prepared at 3 mM (Pi3) and 4 mM (Pi4) [55].

### 5.4. PCS and IS Serum Measurement

PCS and IS in patients’ serum were quantified by HPLC as previously described [56]. The supernatant was ultrafiltered with a 30 kDa cutoff membrane Amicon Ultra (Millipore, Burlington, MA, USA). Chromatographic analysis was done using a Shimadzu Prominence system equipped with a Rheodyne injector, a quaternary pump (Shimadzu LC-20AD), controlled by the LC Solution software and a fluorescence detector (Shimadzu RF-20A). The toxins were separated on a C8 Luna column (5 μm, 100 Å, 150 × 4.6 mm) (Phenomenex, Torrance, CA, USA), eluted with 50 mM ammonium formate, pH = 3.0 and a methanol gradient of 35–70% (*v/v*), at a flow rate of 0.7 mL/min. The toxins were detected using fluorescence (PCS: λexc = 265 nm and λem = 290 nm [57] and IS: λexc = 283 nm and λem = 380 nm) [58,59].

### 5.5. Measurement of MCP-1, IL-8, sVCAM-1, and sICAM-1 Serum Concentrations

Serum concentrations of MCP-1, IL-8, sVCAM-1, and sICAM-1 were measured using ELISA [3]. The measurement range was 31.25–2000 pg/mL for MCP-1 and IL-8 and 0.03–2 ng/mL for sVCAM-1 and sICAM-1, calculated against standard curves obtained using the corresponding recombinant molecules.

### 5.6. Endothelial Cell Culture and Treatment

An immortalized human endothelial cell line EA.hy926 (ATCC CRL 2922, Manassas, VA, USA) [60] was cultured in DMEM supplemented with 10% fetal bovine serum (FBS) and 10 mg/mL of penicillin/streptomycin and maintained at 37 °C in a humidified atmosphere containing 5% CO_2_. The cells were treated with uremic toxins (PCS, IS, and Pi) at concentrations previously described. In another set of experiments, 10% of the uremic pool (GI, GII, and GIII) was added in the culture media to mimic the environment found in CKD patients.

### 5.7. MTT Cell Viability Assay

The cell density of each well was of 10^4^. After 24 h, the cells were treated for 24 h and then incubated with MTT (3-(4,5-Dimethylthiazol-2-yl)-2,5-diphenyltetrazolium bromide) solution (5 mg/mL) for 4 h. The culture medium was replaced with dimethyl sulfoxide (DMSO) and the absorbance was measured at 570 nm [61]. The results are expressed as % control (non-treated-cells) of 3 experiments done in triplicate.

### 5.8. Cell Permeability Assay

ECs were plated at a density of 2 × 10^5^ cells per well on the top of Transwell^®^ inserts and incubated at 37 °C until confluence. The cells were then treated for 24 h, washed and FITC-Dextran solution (2.5 mg/mL) was added to each insert. After 20 min in the absence of light, an aliquot from the lower compartment was transferred to a 96-well black microplate and the fluorescence was read with Tecan Infinite^®^ 200 PRO microplate reader (Tecan Group Ltd., Männedorf, Switzerland) (λexc = 485 nm and λem = 535 nm). Data were analyzed using the Tecan i-control software Version 1.5.14.0 (Tecan, Salzburg, Austria, 2008) [62]. The insert membranes were then stained with crystal violet for further visualization under an Axiol Imager Z2 light microscope (Carl Zeiss, Jena, Germany). The results were expressed as % control (non-treated-cells). The experiments were performed in quadruplicate.

### 5.9. Scanning Electron Microscopy (SEM)

For SEM, 10^5^ cells were fixed on microscope slides for 1 h in Karnovski solution (2% glutaraldehyde, 4% paraformaldehyde, 1 mM CaCl_2_ in 0.1 M sodium cacodylate buffer), and post-fixed with 1% osmium tetroxide, for 1 h. The cells were dehydrated and then subjected to the critical point in the CPD 010 (Critical Point Dryer), plated with gold in the apparatus SCD 030 (Balzers Union, Liechtenstein). Images were taken under a JEOL JSM 6360–LV Scanning Electron Microscope (Jeol USA Inc., Peabody, MA, USA) [63]. 3 independent experiments were performed in duplicates.

### 5.10. F-actin Staining by Fluorescence Microscopy

10^5^ cells were plated on circular coverslips, treated (24 h) and fixed with 2% PFA. To stain F-actin, two drops of ActinGreen™ 488 ReadyProbes^®^ were added per milliliter of medium. The cells were incubated for 30 min, protected from light. The coverslips were mounted on histological slides with Fluoromount G and DAPI. The cells were observed under a Nikon A1RSiMP+ confocal fluorescence microscope (Nikon Instruments, Tokyo, Japan). 2 independent experiments were performed in duplicates.

### 5.11. Immunochemical Analysis of VE-Cadherin and ZO-1 On Arteries

Another group of patients were included in a separate protocol [64]. During renal transplantation, external iliac and renal artery segments were collected from 13 patients in stage 5 CKD (recipients) and 10 donors (controls) and were immediately fixed in 10% formalin. After specimens were embedded in paraffin and 5 μm sections were prepared. For antigen recovery, the slides were immersed in Immuno Retriever (Dako, Carpinteria, CA, USA) at 99 °C for 25 min. Immunoblotting of VE-cadherin and ZO-1 was done by a Reveal Polyvalent HRP-DAB Detection System (Spring Bioscience Corporation, Pleasanton, CA, USA). The slides were then incubated (12 h) with Anti-human VE-cadherin and ZO-1 used at 2.5 μg/mL and 2.0 μg/mL, respectively, and Spring Reveal Complement and Conjugate. The reaction was developed using a chromogenic substrate DAB (3,3′-diaminobenzidine), and samples were counterstained with hematoxylin. Images were captured under an Axio Imager Z2 microscope (Carl Zeiss, Jena, Germany) and the cell density was quantified using the image analysis software Metafer 4/VSlide (Metasystems, Altlussheim, Germany).

### 5.12. VE-Cadherin and ZO-1 Gene Expression

Total RNA was isolated from the lysed cells using Trizol method [65]. RNA purity and concentration were checked by measuring the A260 nm/A280 nm absorbance ratio on the NanoDrop 2000 spectrophotometer (Thermo Scientific, Waltham, WA, USA). The RNA integrity was analyzed by agarose gel (1%) electrophoresis. The mRNA was transcribed into complementary DNA (cDNA) using the High Capacity RNA-to-cDNA Kit (Applied Biosystems, Foster City, CA, USA). The cDNA was amplified with specific primers (Table 4) and the EvaGreen Master Mix S (Applied Biological Materials, Richmond, BC, Canada) using the Rotor-Gene 6000 thermal cycler (Corbett Research Inc., Mortlake NSW, Australia). The relative gene expression was analyzed using the 2^-ΔΔCT^ method [66,67,68]. The Hypoxanthine Phosphoribosyltransferase (HPRT) was used as a housekeeping gene [69,70,71,72,73,74]. We performed 4 independent experiments in duplicates.

### 5.13. VE-Cadherin and ZO-1 Western Blot Analysis

Approximately 3 × 10^6^ cells were washed with ice-cold PBS and lysed in 100 μL of radioimmunoprecipitation assay buffer (20 mM, Tris-HCl pH 7.5, 150 mM NaCl, 1 mM Na_2_ EDTA, 1 mM EGTA, 2.5 mM sodium pyrophosphate, 1 mM β-glycerophosphate, 1 mM Na_2_PO_4_, 1% NP-40, 1% sodium deoxycholate, and 1 μg/mL leupeptin for 20 min). The total protein concentration was performed using Bradford assay [75]. Equal amounts of protein, (30 μg) were separated using 10% SDS-PAGE and transferred onto polyvinylidene difluoride (PVDF) membranes. The membranes were blocked for 1 h in Tris-buffered saline containing 3% casein and 0.3% Tween 20. After washing with PBS-Tween 20 (0.05%), the membranes were incubated overnight with 1 μg/mL of mouse anti-human VE-cadherin antibody or 2 μg/mL of rabbit anti-human ZO-1 antibody at 4 °C. Primary antibodies were detected using a horseradish peroxidase conjugated-goat anti-mouse IgG (0.01 mg/mL) or anti-rabbit IgG (0.8 mg/mL) antibodies and visualized by enhanced chemiluminescence Western blotting reagents. Band intensity was analyzed using the Software Image Studio Lite 5.0 (Lincoln, NE, USA). 4 independent experiments were performed in duplicates.

### 5.14. VE-Cadherin and ZO-1 Immunofluorescence Analysis

Approximately 10^5^ cells were plated on circular coverslips, treated, and fixed with 2% PFA, followed by washing with PBS and incubation with 0.1% glycine. Subsequently, the cells were incubated with PBS containing 1% BSA and 0.01% saponin for 1 h. The cells were then incubated with 0.5 μg/mL of an anti-human VE-cadherin antibody for 12 h. The coverslips were then washed again and incubated with NorthernLights™ 557 conjugated anti-mouse IgG secondary antibody (1 mg/mL). ZO-1 immunofluorescence analysis followed the same procedure using 2.5 μg/mL of anti-human ZO-1 antibody and Alexa Fluor 488 anti-rabbit antibody (1 mg/mL) in the dark. The coverslips were mounted on histological slides with Fluoromount G and DAPI, sealed with formalin-free colorless enamel and observed under a Nikon A1RSiMP+ confocal fluorescence microscope (Nikon Instruments, Tokyo, Japan). We performed 3 independent experiments in duplicates.

### 5.15. VE-Cadherin and ZO-1 Flow Cytometry Analysis

The cells were treated and released with 2 mM EDTA and scraped. After fixation with PFA 2%, the immunolabeling was performed as described above for immunofluorescence. Twenty thousand events of each sample were acquired using a FACSCalibur (BD Biosciences, San Jose, CA, USA) cytometer, and the CellQuest software (BD Biosciences, CA, USA) were acquired. Data were analyzed using Flowing Software 2.5.1 (Cell Imaging Core, Turku, Finland, 2013). Three independents experiments were performed in duplicates.

### 5.16. Statistical Analysis

The Shapiro-Wilk normality test was used, followed by the Student’s t-test or Anova test to statistically analyze parametric data. For non-parametric data, a Mann-Whitney or Anova on Rank’s tests was used. A *P* < 0.05 value between groups was considered significant. Values were expressed as means ± standard error of the mean (SEM). Data were analyzed using the GraphPad Prism 6.01 statistical package (GraphPad Software Inc., La Jolla, CA, USA, 2012).

## Figures and Tables

**Table 1:** Clinical characteristics of the study population.

Analyzed Parameters	Mean Or Percent
Patients (*n*)	80
Traditional risk factors	
Mean age ± SEM, years	62.7 ± 1.3
Gender, % male	61.0
Race, % caucasians	86.0
Smoking, %	33.0
Alcoholism, %	14.0
*Diabetes mellitus*, %	43.0
Hypertension, %	79.0
Dyslipidemia, %	56.3
Primary kidney disease	
Nephrosclerosis, %	30.0
Diabetic nephropathy, %	21.3
Chronic glomerulonephritis, %	10.0
Polycystic kidney disease, %	12.5
Others and unknown, %	26.2

**Table 2:** Laboratory parameters of the study population.

Analyzed Parameters	Media ± SEM	Range
Biochemical characterization		
Uric acid, mg/dL	7.0 ± 2.0	1.7–15.6
Albumin, g/dL	4.2 ± 0.6	2.2–5.7
Calcium, mg/dL	8.6 ± 3.0	1.0–12.7
Creatinine, mg/dL	2.3 ± 1.5	0.5–7.6
Glucose, mg/dL	116.0 ± 52.1	53.0–284.0
Potassium, mmol/L	5.0 ± 0.6	3.5–7.0
Sodium, mmol/L	141.0 ± 4.5	132.0–158.0
Urea, mg/dL	77.0 ± 40.6	13.0–189.0
PCS, mg/L	20.5 ± 19.0	0.01–92.35
IS, mg/L	4.6 ± 6.5	0.25–40.26
Pi, mg/dL	4.4 ± 1.1	2.4–9.9

PCS: p-cresyl sulfate; IS: indoxyl sulfate; Pi: inorganic phosphate.

**Table 3:** Clinical characteristics and biochemical parameters of the three uremic pools.

Analyzed Parameters	GI (*n* = 7) Latent CKD	GII (*n* = 42) Mild CKD	GIII (*n* = 31) Severe CKD
Traditional risk factors			
Smoking, %	52	36	45
Alcoholism, %	22	12	26
Diabetes mellitus, %	43	32	58
Hypertension, %	78	100	94
Dyslipidemia, %	57	56	58
CVD, %	43	36	55
Biochemical characterization			
Uric acid (mg/dL)	6.1	7.0	7.7
Albumin (g/dL)	4.5	4.2	4.0
Calcium (mg/dL)	9.7	7.7	8.6
Creatinine (mg/dL)	1.0	1.6	3.7
Glucose (mg/dL)	137.1	95.3	120.8
hs-CRP (mg/L)	5.1	5.9	7.4
Potassium (mmol/L)	5.0	5.0	5.0
Sodium (mmol/L)	141.0	142.0	139.0
Urea (mg/dL)	42.3	63.9	114.0
PCS (mg/L)	11.1	15.4	33.9
IS (mg/L)	1.5	2.1	9.6
Pi (mg/dL)	4.1	4.1	5.0

CVD: cardiovascular disease; hs-CRP: high sensitivity C-reactive protein; PCS: p-cresyl sulfate; IS: indoxyl sulfate, Pi: inorganic phosphate.

**Table 4:** Sequence of the specific primers used for gene amplification.

Target Gene	Primers	Amplicon
*VE-cadherin*	5’-CAGCCCAAAGTGTGTGAGAA-3’ (F) 5’-CGGTCAAACTGCCCATACTT-3’ (R)	185 pb
*ZO-1*	5’-GCGGTCAGAGCCTTCTGATC-3’ (F) 5’-CATGCTTTACAGGAGTTGAGACAG-3’ (R)	122 pb
*p120*	5’-GATGCTGTCAAGTCCAATGCAG-3’ (F) 5’-AGTACTGGGATGCCCTTGAGC-3’ (R)	101 pb
*β-catenin*	5’-GTGCTATCTGTCTGCTCTAGTA-3’ (F) 5’-CTTCCTGTTTAGTTGCAGCATC-3’ (R)	154 pb
*Vinculin*	5’-TCAGATGAGGTGACTCGGTTGG-3’ (F) 5’-GGGTGCTTATGGTTGGGATTCG-3’ (R)	109 pb
*HPRT*	5’-GAACGTCTTGCTCGAGATGTGA-3’ (F) 5’-TCCAGCAGGTCAGCAAAGAAT-3’ (R)	101 pb

## Supplementary Materials

The following are available online at http://www.mdpi.com/2072-6651/10/10/404/s1, Figure S1: Methodology flowchart 1, Figure S2: Methodology flowchart 2, Figure S3: Methodology flowchart 3.
