# Supplementary material for: Uremia Impacts VE-Cadherin and ZO-1 Expression in Human Endothelial Cell-to-Cell Junctions
Source: Toxins (Basel). 2018 Oct 7;10(10):404. doi: 10.3390/toxins10100404 (PMC6215219; doi:10.3390/toxins10100404)
Supplement: Supplementary file 1 [file toxins-10-00404-s001.pdf]

Toxins **2018**, *10*, 404; doi: 10.3390/toxins10100404

Supplementary Materials: Uremia Impacts VE-Cadherin and ZO-1 Expression in Human Endothelial Cell-to-Cell Junctions

Rayana A. P. Maciel, Regiane S. Cunha, Valentina Busato, Célia R. C. Franco, Paulo C. Gregório, Carla J. R. Dolenga, Lia S. Nakao, Ziad A. Massy, Agnès Boullier, Roberto Pecoits-Filho and Andréa E. M. Stinghen

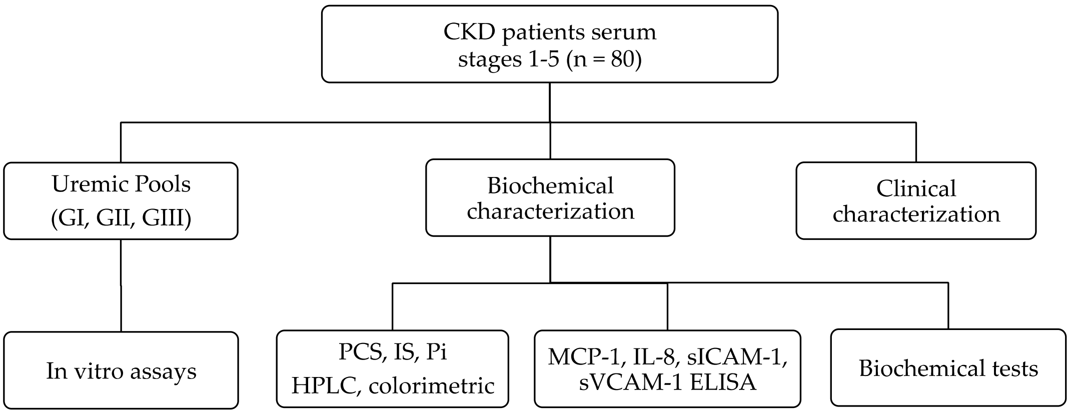

Figure S1. Methodology flowchart 1.

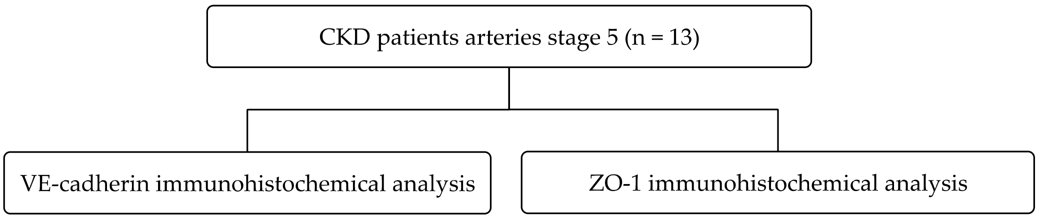

Figure S2. Methodology flowchart 2.

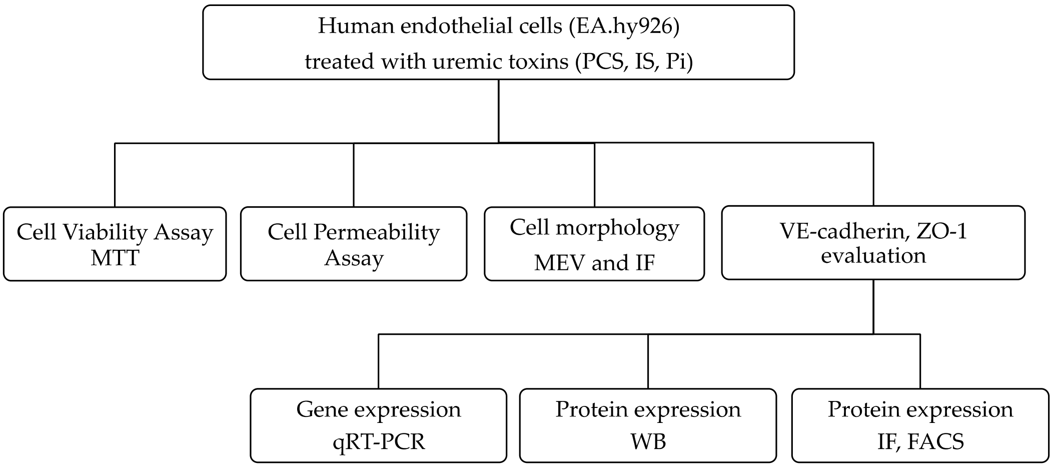

Figure S3. Methodology flowchart 3.
